# The NAC family transcription factor GmNAC42–1 regulates biosynthesis of the anticancer and neuroprotective glyceollins in soybean

**DOI:** 10.1186/s12864-019-5524-5

**Published:** 2019-02-20

**Authors:** Md Asraful Jahan, Brianna Harris, Matthew Lowery, Katie Coburn, Aniello M. Infante, Ryan J. Percifield, Amanda G. Ammer, Nik Kovinich

**Affiliations:** 1 Division of Plant and Soil Sciences, West Virginia University, Morgantown, West Virginia 26506 USA; 2 Department of Biology, West Virginia University, Morgantown, West Virginia 26506 USA; 3 Department of Biochemistry, West Virginia University, Morgantown, West Virginia 26506 USA; 4 Department of Biostatistics, West Virginia University, Morgantown, West Virginia 26506 USA; 5 Microscope Imaging Facility, West Virginia University, Morgantown, West Virginia 26506 USA

**Keywords:** Phytoalexin, Transcription factor, NAC, Isoflavonoids, Glyceollins

## Abstract

**Background:**

Glyceollins are isoflavonoid-derived pathogen-inducible defense metabolites (phytoalexins) from soybean (*Glycine max* L. Merr) that have important roles in providing defense against pathogens. They also have impressive anticancer and neuroprotective activities in mammals. Despite their potential usefulness as therapeutics, glyceollins are not economical to synthesize and are biosynthesized only transiently and in low amounts in response to specific stresses. Engineering the regulation of glyceollin biosynthesis may be a promising approach to enhance their bioproduction, yet the transcription factors (TFs) that regulate their biosynthesis have remained elusive. To address this, we first aimed to identify novel abiotic stresses that enhance or suppress the elicitation of glyceollins and then used a comparative transcriptomics approach to search for TF gene candidates that may positively regulate glyceollin biosynthesis.

**Results:**

Acidity stress (pH 3.0 medium) and dehydration exerted prolonged (week-long) inductive or suppressive effects on glyceollin biosynthesis, respectively. RNA-seq found that all known biosynthetic genes were oppositely regulated by acidity stress and dehydration, but known isoflavonoid TFs were not. Systemic acquired resistance (SAR) genes were highly enriched in the geneset. We chose to functionally characterize the NAC (NAM/ATAF1/2/CUC2)-family TF *GmNAC42–1* that was annotated as an SAR gene and a homolog of the *Arabidopsis thaliana* (Arabidopsis) indole alkaloid phytoalexin regulator *ANAC042*. Overexpressing and silencing *GmNAC42–1* in elicited soybean hairy roots dramatically enhanced and suppressed the amounts of glyceollin metabolites and biosynthesis gene mRNAs, respectively. Yet, overexpressing *GmNAC42–1* in non-elicited hairy roots failed to stimulate the expressions of all biosynthesis genes. Thus, *GmNAC42–1* was necessary but not sufficient to activate all biosynthesis genes on its own, suggesting an important role in the glyceollin gene regulatory network (GRN). The GmNAC42–1 protein directly bound the promoters of biosynthesis genes *IFS2* and *G4DT* in the yeast one-hybrid (Y1H) system.

**Conclusions:**

Acidity stress is a novel elicitor and dehydration is a suppressor of glyceollin biosynthesis. The TF gene *GmNAC42–1* is an essential positive regulator of glyceollin biosynthesis. Overexpressing *GmNAC42–1* in hairy roots can be used to increase glyceollin yields > 10-fold upon elicitation. Thus, manipulating the expressions of glyceollin TFs is an effective strategy for enhancing the bioproduction of glyceollins in soybean.

**Electronic supplementary material:**

The online version of this article (10.1186/s12864-019-5524-5) contains supplementary material, which is available to authorized users.

## Background

In 1939 K.O. Mueller et al. reported that metabolites that were elicited in potato upon inoculation with an incompatible race of *Phytophthora infestans* subsequently provided resistance to a compatible race [1]. Since then, the pathogen-inducible defense metabolites that have been identified from numerous plant species have collectively been referred to as ‘phytoalexins’. Some phytoalexins have essential roles in defending agricultural crops against major pathogens. A classic example is the glyceollins of soybean that provide resistance to the oomycete *Phytophthora sojae* [2–4]. For decades researchers have studied the genetic regulation of phytoalexin elicitation by pathogens. Efforts have recently focused on identifying the transcription factors (TFs) that activate phytoalexin biosynthesis, a goal that has been confounded by the myriad of plant responses that occur synchronously in response to pathogens. Phytoalexins are biosynthetically diverse among plant species and include the isoflavonoid-derived glyceollins from soybean, the phenylpropanoid stilbenes from grapevine, the phenolic aldehyde gossypol from cotton, the terpenoid momilactones and phytocassanes from rice, and the indole alkaloid camalexin from Arabidopsis [5–10]. Since the TFs that activate the biosynthesis of phytoalexins in different plant species belong to different gene families and/or are non-homologous, for decades an important question has remained whether phytoalexin TFs are as diverse as the biosynthetic pathways that they regulate. Yet, several excellent reviews highlight that phytoalexins share common abiotic elicitors [11–13]. This could suggest conserved regulatory pathways and TFs among plant species despite the biosynthetic heterogeneity of phytoalexins.

Highly conserved abiotic elicitors of phytoalexins include heavy metals, herbicides, and UV irradiation. UV elicits stilbene phytoalexins in grapevine, *Cissus Antarctica*, and *Cannabis sativa* [14], the flavonoid and diterpenoid phytoalexins in rice [15, 16], camalexin in Arabidopsis [17], and glyceollins in soybean [18]. In rice, loss-of-function mutants of the JA biosynthesis gene allene oxide cyclase (*aos*) or jasmonic acid-amido synthetase (*osjar1–2*) resulted in an almost complete loss of sakuranetin elicitation in response to UV [19]. Yet, the diterpenoid phytoalexins of rice were not affected in JA biosynthesis mutants. Copper chloride (CuCl_2_) elicitation of sakuranetin, momilactone, and diterpenoid phytoalexins in rice was dramatically reduced by JA biosynthesis inhibitors [20]. The heavy metal silver nitrate (AgNO_3_) elicited glyceollin accumulation in soybean by reducing its degradation and by enhancing the hydrolysis of isoflavone-glycoside conjugates that compete with glyceollins for the common biosynthetic intermediate daidzein [21]. AgNO_3_ was shown to antagonize many plant development processes by inhibiting ethylene perception [22]. Yet, glyceollin elicitation by AgNO_3_ was largely independent of ethylene signaling [21]. Herbicides such as acifluorfen elicit at least in part via the reactive oxygen species (ROS) signaling pathway(s). The *ups1* loss-of-function mutant of Arabidopsis defective in ROS signaling had reduced camalexin levels in response acifluorfen [23]. *ups1* also had reduced camalexin levels in response to *Pseudomonas syringae* and *P. syringae* pv *maculicola* (*Psm*), suggesting a shared biotic and abiotic elicitation pathway. In soybean, treatments with JA, ethylene, *P. sojae* WGE, or hydroxyl radical (a ROS) were highly effective at priming glyceollin biosynthesis in cells distal to the point of treatment, whereas SA was not [23, 24].

In contrast to the abiotic stresses and signaling molecules that have conserved roles in eliciting phytoalexins in response to abiotic stresses, the TFs found to regulate phytoalexin biosynthesis have varied widely among plant species. GaWRKY1 activated gossypol biosynthesis in cotton [8]. *GaWRKY1* transcripts were induced by methyl jasmonate (MeJA) and *Verticillium dahlia* but not by SA or H_2_O_2_. *GaWRKY1* transcripts were co-expressed both spatially and temporally with gossypol biosynthesis genes and GaWRKY1 was able to directly bind the promoter of (+)-δ-*cadinene synthase* (*CAD1*) in the Y1H system. Another WRKY-family TF, namely AtWRKY33, was identified from Arabidopsis to directly bind and activate the promoter of the camalexin biosynthesis gene *PAD3* [25]. WRKY33 transcripts were induced by the ROS-inducing herbicide paraquat, SA, and necrotrophic fungal pathogens [10]. *GaWRKY1* and *AtWRKY33* were not homologous since the proteins they encode had more than 20 other proteins that were more similar by reciprocal BLASTPs.

The R2R3-type MYB TF genes *VvMYB14* and *VvMYB15* from grapevine were co-induced with stilbene biosynthesis genes in response to UV irradiation, wounding, and the pathogen *Plasmopara viticola* [26]. The proteins directly bound the promoter of *STILBENE SYNTHASE* (*STS*) in transient gene reporter assays using grapevine suspension cells and induced the accumulation of stilbenes when overexpressed in grapevine hairy roots [26]. Homologs of *VvMYB14* and *VvMYB15* in Arabidopsis did not regulate camalexin biosynthesis but rather cold tolerance and defense-induced lignification, respectively [27, 28]. Double and triple mutants of the Arabidopsis R2R3 MYBs *AtMYB34*, *AtMYB51*, and *AtMYB122* had reduced camalexin levels upon elicitation with UV, AgNO_3_, and a PAMP isolated from *Pythium aphanidermatum* (*PaNie*) [29]. However, these three MYBs were unable to bind camalexin biosynthesis gene promoters and feeding the triple mutant plant with a biosynthetic intermediate restored camalexin accumulation, suggesting that *AtMYB34*, *AtMYB51*, and *AtMYB122* did not regulate camalexin biosynthesis directly but rather an upstream process in the elicitation pathway [29]. The constitutive overexpression of the sorghum R2R3 MYB gene *yellow seed* (*y1*) in maize resulted in the ectopic accumulation of 3-deoxyanthocyanidins in vegetative tissues only upon challenge with *Colletotrichum graminicola* [5]. *VvMYB15* and *VvMYB14* were not homologs of *y1* since reciprocal BLASTp’s revealed 5–20 proteins that were more similar.

RNAi silencing of the bHLH-family TF gene *OsMYC2* from rice almost completely eliminated the elicitation of sakuranetin in response to JA treatment [6]. OsMYC2 directly activated the promoter of a sakuranetin biosynthesis gene by transient transactivation assays in rice leaves [6]. Transcripts of another bHLH TF gene from rice, namely *OsDPF*, were inducible in rice leaves by UV, CuCl_2_ and blast infection [9]. OsDPF directly activated the promoters of phytocassane and momilactone biosynthesis genes by transient transactivation assays in rice leaves. Overexpressing *OsDPF* resulted in increased expression of all diterpenoid biosynthetic genes and the accumulation of momilactones and phytocassanes, whereas decreased levels were observed in RNAi knock-down lines. Two homologous JA-inducible bHLHs, TSAR1 and TSAR2, were identified to directly activate triterpene saponin biosynthesis genes in *Medicago truncatula* [9]. TSAR1 and TSAR2 were not among the top 20 most similar proteins compared to OsDPF or OsMYC2, and OsDPF was only the 10th most similar to OsMYC2.

A NAC-type TF gene, *AtANAC042*, was identified from Arabidopsis by T-DNA insertion mutagenesis to have reduced levels of camalexin biosynthesis gene expressions and metabolites when elicited with the ROS-inducing herbicide acifluorofen, bacterial flagellin, or *A. brassicicola* [7]. Bacterial flagellin stimulated the accumulation of *AtANAC042* transcripts at the elongation zone of the root (the site of camalexin biosynthesis), and the induction was abolished in the presence of either MeJA, a general kinase inhibitor (K252a), or a Ca^2+^-chelator (BAPTA).

Collectively, these studies have demonstrated that phytoalexin biosynthetic pathways are regulated by disparate, non-homologous TFs in different plant species, raising the question of whether any TF has a conserved role in regulating the biosynthesis of phytoalexins in plants. Here, we used a comparative transcriptomics approach on soybean that was exposed to novel abiotic stresses and identified a conserved phytoalexin regulator.

## Materials and methods

### Chemicals

(−)-Glyceollin I was from Dr. Paul Erhardt (University of Toledo). Soybean isoflavonoid standards were purified and characterized according to [21]. Isoflavone standards were from Extrasynthese (France). Solvents were LC-MS grade (Fisher).

### Plant materials and growth conditions

Soybean seeds were obtained from the USDA-GRIN soybean germplasm collection and from Elroy Cober (Agriculture and Agri-Food Canada). Harosoy 63 seeds (16 per batch) were sterilized in 30 mL of 70% ethanol, 0.2% triton X (*v*/v) for 5 min on a mixer wheel, rinsed thrice with sterile water, and imbibed overnight. The imbibate was then discarded to remove growth inhibitors and seeds were transferred to water soaked sterile vermiculite (250 mL in volume) in 500 mL beakers. The beaker tops were covered with ring-shaped sterile cheese cloth and covered with plastic wrap to ensure aseptic growth. The cheese cloth permitted passage of air between plastic wrap and the beaker top and the ring shape permitted the passage of light from above the beaker. Seedlings were grown at 22 °C under a 16 h photoperiod using cool white T5 fluorescent lights (500 μE m^− 2^ s^− 1^). At the first trifoliate leaf stage (~ 8 day old), seedling roots were gently rinsed with sterile water to remove vermiculite and were transferred to stress treatments.

### Stress treatments

For all stress treatments, the roots of five seedlings were wrapped together in a germination paper (Sartorius AG, Göttingen, Germany) saturated with half-strength Murashige and Skoog (MS) medium (pH 5.8) containing vitamins and 1% (*w*/*v*) sucrose unless indicated otherwise. The wrapped seedlings were transferred to a 100 mL beaker containing 50 mL of the above medium for the control, cold, heat, wounding and UV-C treatments. Each of the 100 mL beakers were then placed inside a sterile 500 mL beaker and the 500 mL beaker tops were again covered with a ring-shape cut of sterile cheese cloth overlaid with plastic wrap. The volume of the medium in the basin of the 100 mL beaker was maintained daily for all treatments, with the exception of the dehydration treatment. For dehydration, the medium-saturated germination paper was allowed to dry gradually in the 100 mL beaker containing no medium. All seedlings were grown under the temperature and lighting conditions listed above unless otherwise indicated. For heat and cold treatments, the 500 mL beakers were transferred to 37 and 15 °C, respectively. For high carbon stress, the growth medium in the 100 mL beaker was replaced with 3% sucrose in water. For flooding, control medium was maintained up to the level of the hypocotyl-root junction throughout the 9 d treatment. For phosphate deprivation (−P), half-strength MS medium (pH 5.8) that lacked phosphate was used (Caisson Labs, Smithfield, UT). For UV-C treatment, seedlings in beakers were exposed to a 30 W g30 t8 germicidal light (Philips, NV) every day for 1 h. For acidity stress, seedlings were transferred half-strength MS medium pH 3.0 (acidified with HCl).

After 9 d of treatment (unless indicated otherwise), the five seedlings per treatment were unwrapped and separated, flash-frozen in liquid nitrogen, lyophilized to dryness, and individually ground to a fine powder and stored at − 80 °C for metabolite and RNA extractions. The stored tissue powder was lyophilized again for 1 h prior to weighing.

For hairy root experiments, only secondary roots that grew to 3–6 cm on selection media were considered transgenic and were used for WGE treatments. Roots were cut into 1-cm pieces then overlaid with sterile water (mock) or wall glucan elicitor (WGE) that was extracted from *P. sojae* according to [21]. For RNA extraction, 100 mg of fresh tissue was harvested on ice and freeze dried prior to storage at − 80 °C. For metabolite analyses, fresh hairy root tissues (~ 100 mg) were extracted immediately upon harvesting without lyophilization.

### Isoflavonoid analysis

For analysis of seedlings, lyophilized tissue powder (12 mg) was extracted with 80% ethanol (10 μL mg^− 1^ dry tissue) and isoflavonoid identifications were done by UPLC-PDA-MS^n^ as indicated in [21]. Four seedlings per treatment were individually extracted for metabolite analysis. Metabolite analyses of pH 3.0 medium, dehydration stress, and control treatments were confirmed by three independent experiments.

Hairy roots were extracted with 80% ethanol (1 μL mg^− 1^ fresh weight, FW) as described [21]. For all hairy root experiments, five biological replicates were analyzed per treatment. Two independent transformation experiments were analyzed per DNA construct. Absolute amounts of isoflavonoids were determined by comparison of the UPLC-PDA peak areas to a concentration curve of purified or authentic standards as described in [21].

### RNA extraction and qRT-PCR

Total RNA was isolated from lyophilized tissue powder using the Spectrum Plant Total RNA Kit (Sigma-Aldrich, St. Louis, MO, USA) as described [21]. Total RNA (500 ng) was treated with DNase I (Amplification grade, Invitrogen, Carlsbad, CA, USA) to remove genomic DNA and cDNA was synthesized using SuperScript II Reverse Transcriptase (Invitrogen). cDNA templates were diluted 4-fold with water and qRT-PCR was conducted as described [21]. All qRT-PCR experiments included four biological replicates and two technical replicates. Primers used in this study are listed in Additional file 1: Table S1.

### RNA-seq

Total RNA was extracted from the powder of individual seedlings as described above. Three individual seedlings per stress treatment and their respective controls were used to make a total of 12 libraries for RNA-seq analysis. RNA samples were sent to the Genomics Core Facility of West Virginia University for library preparation. The quality of each RNA sample was determined using an RNA Nano 6000 Chip and an Agilent 2100 Bioanalyzer (Santa Clara, CA). RNA samples with an Integrity Number (RIN) greater than 8.0 were used to prepare the libraries. Following quantification of RNA using a Qubit fluorometer, libraries were constructed from 750 ng using the mRNA stranded library prep kit (KAPA Biosystems) as per manufacturer’s protocol with nine cycles of PCR. The completed cDNA libraries were quantified using a Qubit and pooled in equimolar ratios prior to sequencing at the Marshall University Genomics Core. The 100 bp paired-end reads were generated using a HiSeq1500 system (Illumina). Eight libraries were sequenced per lane in high-output mode.

Data filtering was carried out to eliminate adapter sequences and/or low-quality reads. The quality of raw reads was determined using FastQC software (http://www.bioinformatics.babraham.ac.uk/projects/fastqc/) and clean reads were then mapped/aligned to *Glycine max* reference genome (Gmax_275_V2.0.fa, https://phytozome.jgi.doe.gov/pz/portal.html) using STAR RNA-seq aligner [30] with default mode based on the current gene annotation. Only the paired mapped reads were considered for further analyses. Reads were quantified using using featureCounts [31]. Differentially expressed genes (DEGs) were identified using a Negative Binomial Distribution in DESeq2 [32]. Multiple hypothesis correction was conducted with Benjamini Hochberg procedure to get an adjusted *P* value at 0.05 which decrease the false discovery rate (FDR). Principle component analysis, heatmap and clustering of the samples were done to check the robustness of the analysis. For the identification of gene homologs, genes were considered to be homologous if their predicted protein sequences were the best matches in reciprocal BLASTPs.

### Cloning

The *GmNAC42–1* ORF was PCR amplified from the cDNA of Harosoy63 seedlings treated with pH 3.0 medium (9 dat) by the *attB* Adapter PCR protocol (Invitrogen, Carlsbad, CA) using Phusion polymerase (Thermo Fisher Scientific) and primers (Additional file 1: Table S1). The amplicon was cloned into the donor vector pDONR221 using BP Clonase II (Invitrogen, Carlsbad, CA) and following sequencing was LR recombined downstream of GFP in the pGWB6 vector to assay subcellular localization and downstream of the GAL4 activation domain in the pDEST-GADT7 vector for Y1H. For silencing, a 227-bp region of exon 2 of *GmNAC42–1* was amplified from cDNA and BP cloned into pDONR221, which after sequencing was LR subcloned into the RNAi vector pANDA35HK. Hairpin integrations were confirmed by sequencing.

### Soybean hairy roots

Transgenic soybean hairy roots were produced according to [33] with some modifications. Relatively large Williams 82 soybean seeds without cracks were surface sterilized with 70% isopropyl alcohol (*v*/v) for 30 s and 10% commercial bleach (6.0% (v/v) sodium hypochlorite) for 5 min with gentle agitation, then rinsed three times in sterile MilliQ-filtered water (EMD Millipore, MA). Seeds were transferred to germination paper saturated with germination and co-cultivation (GC) medium (half-strength MS salts (Caisson Labs, UT), 1% sucrose, pH 5.8, and MS vitamins) in a sterile Petri dish and germinated for 3 d in the dark, then transferred to cool white T5 fluorescent lights (100 μE s^− 1^ m^2^) at 24 °C, a condition that was used for all subsequent soybean transformation steps.

Following pre-culture on LB-agar plates containing 50 mg L^− 1^ kanamycin and hygromycin, *Agrobacterium rhizogenes* strain K599 containing the empty vector or construct DNA were resuspended to an OD600 of 0.5–0.8 in phosphate buffer (0.01 M Na_2_HPO_4_, 0.15 M NaCl, pH 7.5) containing 100 μM acetosyringone. Cotyledons were gently twisted off of 6–7 d old seedlings. The apical meristem and hypocotyl was excised and several 1 mm-deep cuts were made across the adaxial surface of the cotyledon with a scalpel previously dipped in the *Agrobacterium* solution. Twenty-four to 36 cotyledons were inoculated per DNA. Cotyledons were placed adaxial-side-down on germination paper saturated with GC medium containing 100 μM acetosyringone and co-cultivated for 3 d at 22 °C under low light (65 μE s^− 1^ m^2^) on a 16 h photoperiod. Cotyledons were then cultured adaxial-side-up on hairy root growth (HRG) medium (half strength MS salts, 3% sucrose (*w*/*v*) (pH 5.8) with gelzan (2.4 g L^− 1^; Sigma-Aldrich, MO), MS vitamins (2.5 mL L^− 1^) and timentin (500 mg L^− 1^). Fourteen to 21 d later, transgenic primary roots with 2–3 cm secondary roots were transferred to and selected on HRG containing 50 mg L^− 1^ kanamycin and hygromycin. Only secondary roots that grew to 3–6 cm were considered transgenic and were used for treatments. All hairy root experiments were conducted two times independently, representative results are shown.

### Subcellular localization

Soybean hairy roots transformed with *nGFP-pGWB6* or *nGFP-NAC42–1-pGWB6* were harvested and stained with propidium iodide according to the manufacturer’s instructions (Sigma-Aldrich, St. Louis, MO, USA). Three-to-four roots per genotype per two independent transformation events were analyzed and a representative result is shown. Confocal images were acquired using a Nikon A1R Si confocal laser with N-SIM-E, a TiE inverted research microscope, and NIS Elements software. Imaging was performed using an Apo oil 60× objective, plus 1.5× optical zoom, and 6× digital zoom. Excitation and emission spectra were 488 nm and 500–550 nm for GFP and 488 nm and 570–620 nm for propidium iodide, respectively.

### Yeast one-hybrid

*G4DT* and *IFS2* promoter regions 1 and 2 flanked by *attL4* and *attR1* recombination sites (Additional file 2: Table S2) were synthesized by Genscript (Piscataway, NJ) and recombined into the destination vector pMW#2 (Addgene, Cambridge, MA) using LR clonase (Invitrogen, Carlsbad, CA). Clones were selected by colony PCR then sequenced. Constructs were linearized by digestion with *AflII* (Thermo Scientific, Waltham, MA) prior to transformation into yeast strain YM4271 (*MATa*, *ura3–52*, *his3–200*, *lys2–801*, *ade2–101*, *ade5*, *trp1–901*, *leu2–3*, *112*, *tyr1–501*, *gal4D*, *gal80D*, *ade5::hisG*) and were selected by growth in dropout medium lacking histidine (SD-His). Bait strains containing genomic integrations were confirmed by colony PCR using a pair of promoter- and genome-specific primers (Additional file 1: Table S1). Bait strains were transformed with pDEST-GADT7 (ABRC, Columbus, OH) or with *GmNAC42–1*-pDEST-GADT7 and transformants were selected on media lacking histidine and leucine (SD-His-Leu) then confirmed by colony PCR. Autoactivation was tested for and positive DNA-protein interactions were determined by growth in SD-His-Leu medium containing increasing concentrations (5, 10, 20, 40 and 60 mM) of 3-amino-1,2,4-triazole (3-AT; Fisher Scientific, Hampton, NH), as previously described [34]. Three biological replicates are shown, results were confirmed by two independent experiments.

## Results

### Novel abiotic stresses that regulate glyceollin biosynthesis

**Fig. 1:**
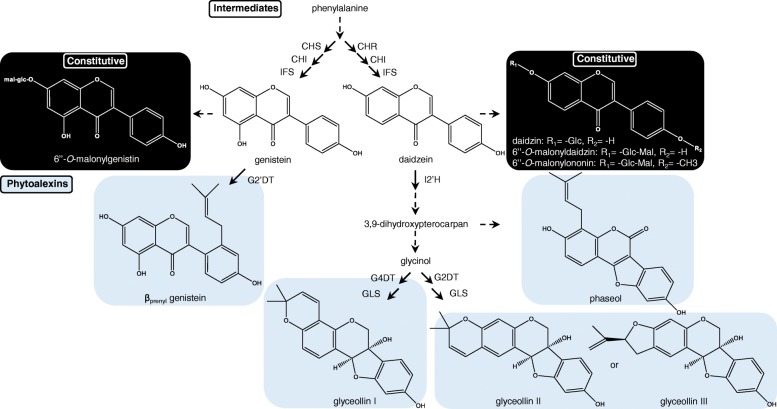
Soybean isoflavonoid biosynthetic pathway. Inducible phytoalexins (highlighted in blue) and constitutively biosynthesized isoflavone conjugates (highlighted in black) are derived from the isoflavone intermediates daidzein or genistein. *CHS* chalcone synthase, *CHR* chalcone reductase, *CHI* chalcone isomerase, *IFS* isoflavone synthase, *G2’DT* genistein 2′-dimethylallyl transferase, *I2’H* isoflavone 2′-hydroxylase, *G4DT* glycinol 4-dimethylallyl transferase, *G2DT* glycinol 2-dimethylallyl transferase, *GLS* glyceollin synthase

To gain insight into how abiotic stresses regulate glyceollin biosynthesis in soybean we first searched for a control growth condition that would allow us to measure the inductive and suppressive effects of abiotic stress treatments on glyceollin biosynthesis. We grew soybean seedlings under two light intensities, 10 and 500 μmol m^− 2^ s^− 1^, which we refer to here as low and high light, respectively. We also compared seedlings grown on soil to those grown in liquid half-strength Murashige and Skoog (MS) medium that can be readily manipulated to provide nutrient and chemical stresses (see Methods). In addition to glyceollins, we also measured the levels of two key biosynthetic intermediates, two additional phytoalexins that have potent anti-pathogenic and/or medicinal activities, and two constitutively biosynthesized isoflavone-glycoside conjugates known to compete with glyceollins for biosynthetic intermediates. Specifically, we measured the levels of glyceollin I, glyceollin II, glyceollin III, and phaseol that are biosynthesized from the intermediate daidzein, and β_prenyl_ genistein that is biosynthesized from genistein (Fig. 1). We also measured the levels of an unknown metabolite that exhibited UV absorbance properties similar to isoflavonoids but did not represent any of the 57 (iso)flavonoid standards that we have in our library.

**Fig. 2:**
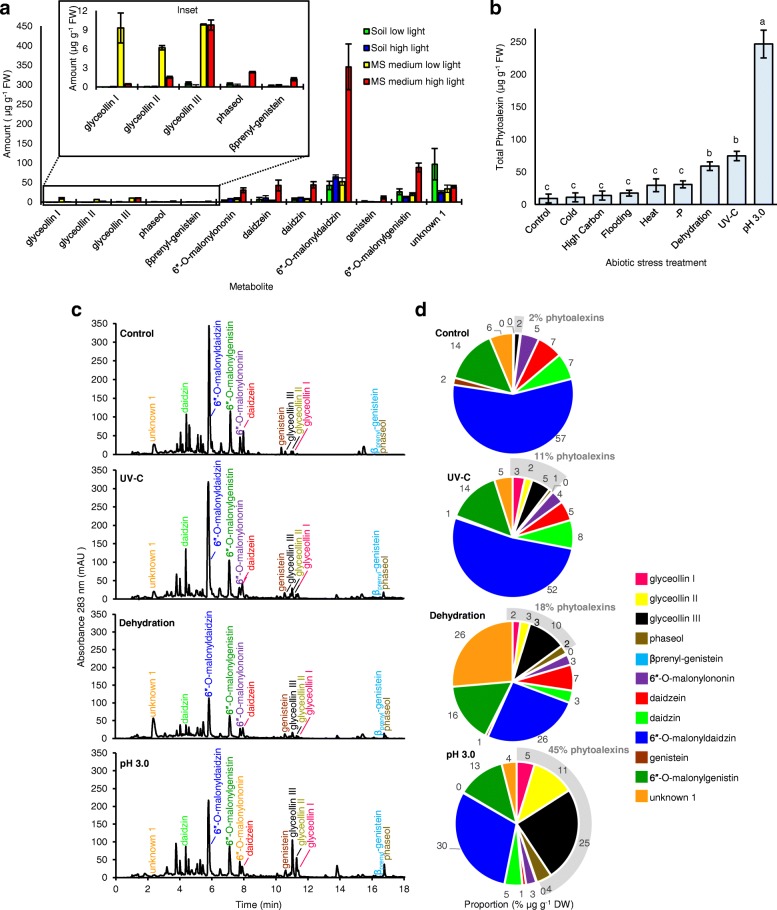
Phytoalexin and isoflavonoid content in response to abiotic stresses. a Effects of light and growth medium on phytoalexin elicitation in Harosoy 63 seedlings. Error bars represent standard error of mean. **b** Effects of different abiotic stress treatments phytoalexin elicitation. Total phytoalexins represent the sum of glyceollins I-II, phaseol, and βprenyl-genistein. Different letters show significant differences by single factor ANOVA, Tukey post hoc test, *P* < 0.01. Error bars represent standard error of mean. **c** UPLC-PDA chromatograms of ethanolic extracts at 283 nm. **d** Proportions of the metabolites labeled in C based on their tissue concentration (μg g^− 1^ DW)

The MS medium high light condition was the only condition that elicited measurable amounts of all phytoalexins (Fig. 2a). The MS low light condition had greater amounts of glyceollins I and II but lacked phaseol and B_prenyl_-genistein, and thus may not be suitable for evaluating the specificity of the effects of abiotic stresses on the glyceollin pathway. Glyceollins were absent or in trace amounts in seedlings grown on soil, either under the high or low light conditions. Based on these results, we selected the MS medium high light as the control condition to evaluate the effects of abiotic stresses on glyceollin biosynthesis.

Seedlings were transferred to eight abiotic stress conditions and the amounts of total phytoalexins were enhanced significantly by pH 3.0 medium, UV-C, and dehydration compared to the control (ANOVA, Tukey post hoc test, *P* < 0.01) (Fig. 2b). pH 3.0 medium stimulated the greatest increase, having 22.7-fold greater amounts of total phytoalexins compared to the control and significantly greater amounts compared to all other treatments. UPLC-PDA chromatograms revealed major increases in the levels of glyceollins for pH 3.0 medium, and major reductions in the amounts of 6-*O*-malonyldaidzin for dehydration and pH 3.0 medium that were not observe for the UV-C treatment (Fig. 2c). pH 3.0 medium and dehydration predominantly caused increases in the amounts of glyceollin III and glyceollin II (Fig. 2d). Overall, pH 3.0 medium had the greatest increase in glyceollin amounts, with glyceollin III becoming 25% of the total measured isoflavonoid content.

### Acidity stress enhances and dehydration suppresses glyceollin biosynthesis

Pathogens generally elicit maximum glyceollin biosynthesis within 24–48 h of inoculation, then the levels rapidly decline [4, 35]. To understand the dynamics of the regulation of glyceollin biosynthesis by pH 3.0 medium and dehydration, we measured metabolite levels at regular intervals up to 9 dat.

**Fig. 3:**
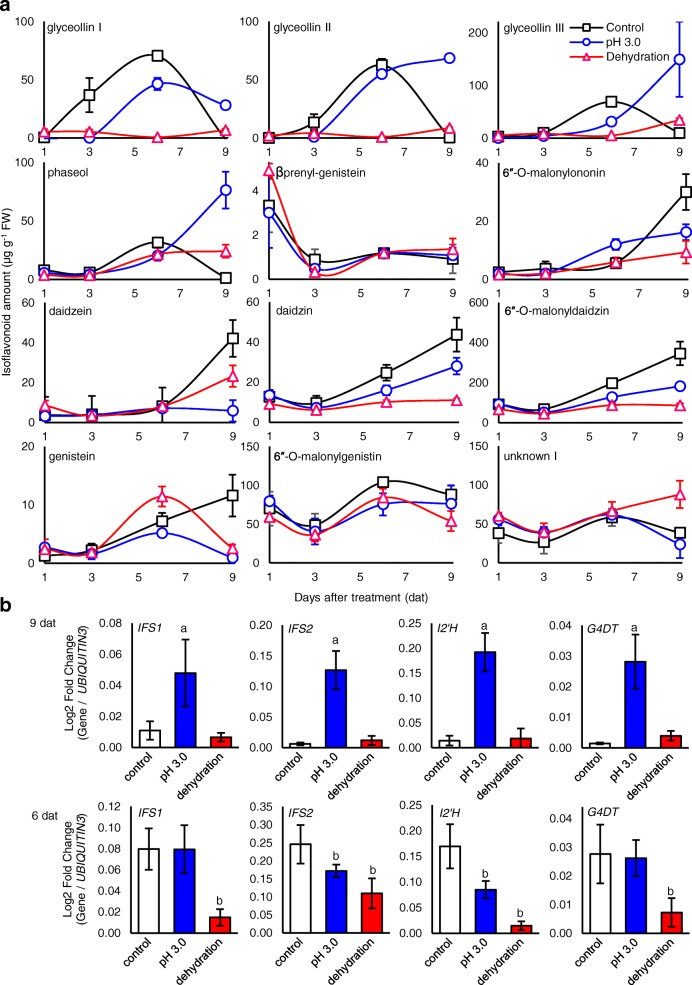
Time course of phytoalexin and isoflavonoid biosynthesis during acidity and dehydration stresses. **a** Isoflavonoid levels by UPLC-PDA over time after transfer to the control condition, pH 3.0 medium, or dehydration stress. Error bars represent standard error of mean. **b** Isoflavonoid biosynthesis gene expressions at 6 and 9 dat measured by qRT-PCR. ^a^Significantly greater and ^b^significantly less than control, paired students *t*-test (*P* < 0.01). Error bars represent standard error of mean

Following the transfer of seedlings to the control condition, we observed a gradual accumulation of glyceollins and phaseol peaking at 6 dat (Fig. 3a). In contrast, β_prenyl_-genistein rapidly decreased up to 3 dat then remained constant thereafter. Two elicitation patterns distinguished pH 3.0 medium from the control. Glyceollin III and phaseol exhibited sharp increases from 6 dat to 9 dat, whereas glyceollins I and II exhibited delayed and prolonged accumulation (Fig. 3a). Elicitation of these daidzein-derived phytoalexins was accompanied by decreases in daidzein and its glycosyl-conjugates, namely daidzin and 6-*O*-malonyldaidzin. Genistein and derived isoflavonoids were not increased by pH 3.0 medium. In sharp contrast, dehydration caused a sustained suppression of all daidzein-derived isoflavonoids over the 9 d period with up to a 106.8-fold suppression of glyceollin I at 6 dat (Fig. 3a). This major suppressive effect was not observed for genistein-derived metabolites.

To determine whether pH 3.0 medium and dehydration stresses regulated glyceollin biosynthesis gene transcripts, we measured the expression of key biosynthetic genes by quantitative reverse transcriptase-polymerase chain reaction (qRT-PCR). Specifically, we measured the expressions of isoflavone synthase 1 (*IFS1*) and *IFS2*, isogenes for the biosynthesis of isoflavones (Fig. 1). We also measured the expressions of isoflavone 2′-hydroxylase (*I2’H*) and glycinol 4-dimethylallyltransferase (*G4DT*), genes for the biosynthesis of all daidzein-derived phytoalexins and glyceollin I, respectively [36, 37].

pH 3.0 medium upregulated all gene transcripts at 9 dat. The levels ranged from 4.4- to 20.7-fold greater than the control for *I2’H* and *IFS2*, respectively (Fig. 3b). By contrast, dehydration stress had reduced levels of all gene transcripts at 6 dat, ranging from 2.2- to 11.7-fold less than the control for *IFS2* and *I2’H*, respectively.

### Acidity and dehydration stresses oppositely regulate all known glyceollin biosynthesis genes

To investigate whether pH 3.0 medium and dehydration oppositely regulated all known glyceollin biosynthesis genes, we conducted RNA-seq comparing genes upregulated by pH 3.0 medium to those downregulated by dehydration.

**Fig. 4:**
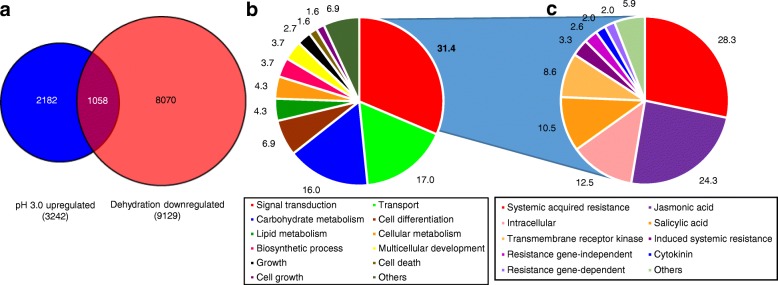
Comparative transcriptomics of seedlings treated with acidity stress or dehydration. **a** Number of genes in Harosoy 63 seedlings that were upregulated and downregulated more than 2-fold by pH 3.0 medium and dehydration, respectively (*P* < 0.05) by RNA-seq. **b** Percent of genes upregulated by pH 3.0 medium and downregulated by dehydration stress assigned to a category of gene ontology. **c** Breakdown of the ‘Signal transduction’ category into gene ontologies. Ontology analysis was conducted using the SoyBase Gene Model Data Mining and Analysis tool

pH 3.0 medium upregulated 3242 and dehydration downregulated 9129 genes more than 2-fold, respectively (*P* < 0.05) (Additional file 3: Table S3 and Additional file 4: Table S4). By comparing the two gene lists, we found that 1058 genes were in common (Fig. 4a & Additional file 5: Table S5). All 27 known glyceollin biosynthesis genes spanning from phenylalanine ammonia lyase (*PAL*) to the glycinol:dimethylallyl diphosphate (DMAPP) transferases *G4DT* and *G2DT* [37, 38] were upregulated by pH 3.0 medium and downregulated by dehydration, respectively (Table 1). Since DMAPP is derived from either the cytosolic mevalonate pathway or the plastidic methylerythritol phosphate (MEP) pathway, we checked our lists for these genes. pH 3.0 and dehydration stresses oppositely regulated genes for all steps of the MEP pathway up to DMAPP formation, whereas no mevalonate genes were differentially regulated (Table 1).

**Table 1:** Glyceollin biosynthesis genes upregulated by pH 3.0 medium and downregulated by dehydration

Pathway	Gene symbol	Enzyme	Wm82.a2 (Glyma 2.0)	pH 3.0 medium		Dehydration	
				Log2 FC	*P*-value	Log2 FC	*P*-value
Phenylpropanoid	*PAL*	phenylalanine ammonia-lyase	Glyma.02G309300	1.49	2.00E-05	−2.15	1.69E-10
			Glyma.03G181700	1.31	0.01535019	−2.08	0.00169183
			Glyma.03G181600/	1.10	0.00697352	−1.83	0.00121303
			Glyma.10G058200				
	*C4H*	cinnamic acid 4-hydroxylase	Glyma.20G114200	4.44	1.63E-170	−1.78	0.00085225
	*4CL*	4-coumarate: coenzyme A ligase	Glyma.11G010500	2.17	2.39E-24	−1.08	0.00147188
	*CHR*	chalcone reductase	Glyma.14G005700	2.03	3.46E-10	−1.64	0.00053532
			Glyma.02G307300	3.78	6.27E-25	−3.25	1.27E-07
	*CHI*	chalcone isomerase	Glyma.10G292200	1.57	3.94E-11	−4.72	4.19E-43
			Glyma.20G241500	1.94	4.17E-19	−1.32	0.02583103
Isoflavonoid	*IFS1*	Isoflavone synthase	Glyma.07G202300	1.97	1.95E-13	−2.12	6.75E-15
	*IFS2*	Isoflavone synthase	Glyma.13G173500	3.23	2.16E-24	−1.11	3.40E-06
	*HIDH*	2-hydroxy-isoflavanone dehydratase	Glyma.10G250300	1.51	2.00E-09	−1.68	1.30E-05
	*I2’H*	isoflavone 2′-hydroxylase	Glyma.15G156100	4.32	1.43E-81	−4.08	7.32E-18
	*IFR*	isoflavone reductase	Glyma.11G070500	2.31	1.52E-29	−4.23	1.34E-11
			Glyma.11G070600	2.67	1.06E-60	−3.28	4.13E-18
			Glyma.01G172600	1.55	1.52E-11	−3.15	4.23E-13
			Glyma.01G172700	1.80	6.88E-13	−2.96	1.54E-20
			Glyma.01G211800	2.48	9.19E-11	−2.56	1.05E-06
			Glyma.11G070200	2.64	1.04E-31	−1.43	9.61E-07
Pterocarpan	*PTS1*	pterocarpan synthase	Glyma.19G151200	3.67	1.31E-20	−2.84	4.06E-06
			Glyma.19G151100	3.78	1.55E-32	−1.53	0.0105246
			Glyma.03G147700	3.75	1.34E-30	−1.70	0.00122376
	*P6αH*	dihydroxypterocarpan- 6α-hydroxylase	Glyma.19G144700	1.71	4.51E-16	−3.79	3.21E-16
	*G4DT*	glycinol 4-dimethylallyl-transferase	Glyma.10G295300	2.93	1.01E-19	−2.91	2.26E-10
	*G2DT*	glycinol 2-dimethylallyl-transferase	Glyma.20G245100	2.80	5.96E-45	−3.11	3.78E-10
Methylerythritol phosphate (MEP)	*DXS*	1-deoxy-D-xylulose 5-phosphate synthase	Glyma.18G148700	2.73	2.90E-32	−4.63	4.62E-22
			Glyma.08G277000	3.15	1.45E-22	−3.33	8.88E-17
			Glyma.08G277100	2.49	1.07E-18	−3.01	5.41E-09
	*DXR*	1-deoxy-D-xylulose 5-phosphate reductoisomerase	Glyma.16G089000	1.07	3.73E-10	−2.68	4.07E-07
			Glyma.17G089600	1.49	6.09E-20	−2.34	2.36E-08
			Glyma.05G037500	1.18	1.29E-23	−1.95	1.59E-07
	*CMK*	4-diphosphocytidyl-2-C-methyl-D-erythritol kinase	Glyma.20G046800	1.33	1.09E-06	−2.06	1.27E-10
	*MDS*	2-C-methyl-D-erythritol 2,4-cyclodiphosphate synthase	Glyma.11G021400	1.10	4.90E-11	−1.46	6.26E-05
	*HDS*	4-hydroxy-3-methylbut-2-enyl diphosphate synthase	Glyma.13G326400	1.82	9.17E-34	−1.19	1.63E-09
	*HDR*	4-hydroxy-3-methylbut-2-enyl diphosphate reductase	Glyma.11G120900	1.90	3.48E-25	−1.16	1.35E-10
			Glyma.12G046000	1.22	6.42E-15	−1.09	1.61E-05
	*IDI2*	IPP isomerase	Glyma.18G242300	1.55	8.00E-07	−1.10	1.05E-06

Since our RNA-seq analyses found that pH 3.0 medium and dehydration regulated glyceollin biosynthesis at the level of transcription, we hypothesized that TF genes required for the activation of those biosynthesis genes would also be present in our geneset. Yet, all previously identified isoflavonoid TF genes were not found. Those absent included TF genes identified by QTL mapping of isoflavonoid amounts, namely *GmMYBJ3* (Glyma.06 g193600) or *GmMYB29* (*Glyma20g35180)* [39, 40]. Also absent were TFs that activated the biosynthesis of chalcone synthase-derived isoflavonoids during seed development, namely *GmMYB176* (Glyma.05G032200) and *GmCYP1* (Glyma.11G098700) [41, 42].

### Comparative transcriptomics identifies candidate transcription factors for the regulation of glyceollin biosynthesis

To better understand the pathways that were oppositely regulated by acidity and dehydration stresses, we analyzed the ontologies of the 1058 oppositely regulated genes (Fig. 4a). Signal transduction was the most common category of ontology (31.4% of genes, Fig. 4b). When the signal transduction category was broken down into ontologies, the greatest proportion (28.3%) were annotated as systemic acquired resistance (SAR) (Fig. 4c). SAR is a component of the plant immune system whereby tissues distant from a pathogen infection site become primed (sensitized) to more rapidly activate resistance responses the second time the plant encounters the pathogen. Gene ontology (GO) enrichment analysis indicated that SAR genes were significantly enriched (*P* < 1.0^− 10^) and included those involved of salicylic acid (SA)-dependent and independent signaling pathways, in addition to jasmonic acid (JA) and ethylene signaling pathways (GO:0009627, GO:0009862, GO:0009864, GO:0009871, and GO:0010112). The SAR genes included homologs of *AGD2-LIKE DEFENSE RESPONSE PROTEIN 1* (*ALD1*) and *FLAVIN-DEPENDENT-MONOOXYGENASE1* (*FMO1*) that were indispensable for SAR in Arabidopsis (Table 2) [43–45]. *ALD1* encodes an enzyme that synthesizes the non-protein amino acid pipecolic acid (Pip) from Lys upon pathogen attack [45]. FMO1 converts Pip to N-hydroxypipecolic acid (NHP) [46] and is needed for Pip to orchestrate priming of pathogen responses by SA-dependent and independent pathways [47]. The SAR genes also included homologs of signaling and TF genes that had roles in regulating the elicitation of the indole alkaloid phytoalexin camalexin in Arabidopsis. *PHYTOALEXIN DEFICIENT4* (*PAD4*) is a lipase-like gene required for SA-dependent elicitation of camalexin in response to microbial pathogens [48]. *SIGMA FACTOR BINDING PROTEIN 1* (*SIB1*) encodes a TF that activates the expression of *AtWRKY33*, a direct regulator of camalexin biosynthesis genes [49]. However, homologs of *AtWRKY33* (namely Glyma.02G232600 and Glyma.14G200200) were not found in our gene set nor were they significantly upregulated by pH 3.0 medium alone.

**Table 2:** Select SAR genes upregulated by pH 3.0 medium and downregulated by dehydration

Accession (Wm82.a2.v1)	Arabidopsis gene symbol	Annotation	Arabidopsis accession	pH 3.0 medium		Dehydration		BLASTP score	E-value (E < 10^−6^)
				Log2 FC	*P*-value	Log2 FC	*P*-value		
Glyma.08G180600	*ALD1*	*AGD2-LIKE DEFENSE RESPONSE PROTEIN 1*	AT2G13810	3.848	0.000	−6.507	0.000	0.0E+ 00	100
Glyma.17G046600	*FMO1*	*FLAVIN-DEPENDENT MONOOXYGENASE 1*	AT1G19250	6.575	0.000	−3.981	0.000	0.0E+ 00	100
Glyma.06G156300	*PAD4*	*PHYTOALEXIN DEFICIENT 4*	AT3G52430	1.809	0.000	−1.753	0.001	0.0E+ 00	100
Glyma.03G249100	*SIB1*	*SIGMA FACTOR BINDING PROTEIN 1*	AT3G56710	4.133	0.000	−5.521	0.000	6.0E-90	100
Glyma.04G134200				2.034	0.000	−1.140	0.007	6.0E-89	100
Glyma.02G284300	*ANAC042/JUB1*	*NAC DOMAIN CONTAINING PROTEIN 42/JUNGBRUNNEN 1*	AT2G43000	1.178	0.000	−2.568	0.000	0.0E+ 00	100
Glyma.18G110700				1.846	0.000	−1.527	0.003	0.0E+ 00	100
Glyma.14G030700				3.029	0.000	−1.197	0.011	0.0E+ 00	100
Glyma.13G267700	*WRKY70*	*WRKY DNA-BINDING PROTEIN 70*	AT3G56400	4.660	0.000	−3.175	0.005	0.0E+ 00	100
Glyma.13G267500				3.029	0.000	−5.123	0.000	0.0E+ 00	100
Glyma.18G213200				1.086	0.000	−4.386	0.000	0.0E+ 00	100
Glyma.04G061400	*WRKY40*	*WRKY DNA-BINDING PROTEIN 40*	AT1G80840	2.140	0.000	−2.580	0.000	1.0E-162	100
Glyma.14G103100				1.712	0.000	−5.104	0.000	0.0E+ 00	100
Glyma.11G120400	*DIR1*	*DEFECTIVE IN INDUCED RESISTANCE 1*	AT5G48485	1.327	0.000	−4.214	0.000	2.0E-68	100
Glyma.12G045500				1.220	0.002	−3.198	0.000	2.0E-67	100
Glyma.06G137000	*DMR6*	*DOWNY MILDEW RESISTANT 6*	AT5G24530	1.428	0.000	−4.998	0.000	0.0E+ 00	100
Glyma.14G048900	*EFE*	*ETHYLENE FORMING ENZYME*	AT1G05010	2.006	0.000	−1.333	0.017	0.0E+ 00	100
Glyma.08G128900	*EFR*	*EF-TU RECEPTOR*	AT5G20480	2.574	0.000	−2.045	0.007	0.0E+ 00	100
Glyma.07G103700	*YLS9*	*NDR1/HIN1-LIKE 10*	AT2G35980	3.041	0.000	−3.884	0.000	2.0E-148	100
Glyma.07G103800				1.466	0.001	−2.917	0.002	8.0E-152	100
Glyma.09G003100	*RLK1*	*RECEPTOR-LIKE PROTEIN KINASE 1*	AT5G60900	2.462	0.000	−2.888	0.000	0.0E+ 00	100
Glyma.03G090200				2.254	0.000	−1.321	0.000	0.0E+ 00	100
Glyma.15G258400				1.753	0.012	−4.378	0.000	0.0E+ 00	100
Glyma.16G168700	*RLP19*	*RECEPTOR LIKE PROTEIN 19*	AT2G15080	3.743	0.000	−5.408	0.000	0.0E+ 00	99.61
Glyma.16G169500	*RLP32*	*RECEPTOR LIKE PROTEIN 32*	AT3G05650	1.890	0.002	−2.689	0.001	0.0E+ 00	100
Glyma.16G170700				2.118	0.023	−3.478	0.001	0.0E+ 00	98.59
Glyma.16G175100	*RLP33*	*RECEPTOR LIKE PROTEIN 33*	AT3G05660	6.601	0.000	−5.803	0.000	0.0E+ 00	100
Glyma.14G046000				3.548	0.000	−1.017	0.025	0.0E+ 00	99.76
Glyma.16G126100	*RLP46*	*RECEPTOR LIKE PROTEIN 46*	AT4G04220	2.151	0.017	−2.953	0.022	0.0E+ 00	93.3

Among the putative soybean SAR genes were three homologs of the NAC [no apical meristem (NAM), Arabidopsis transcription activation factor [*ATAF1/2*] and cup-shaped cotyledon (*CUC2*)] family gene *ANAC042*/*AtJUB1*. *ANAC042*/*AtJUB1* regulates camalexin biosynthesis in Arabidopsis in response to the ROS-inducing herbicide acifluorofen, *Alternaria brassicicola*, and bacterial flagellin (Flg22) [7].

### *NAC42*-type TFs are upregulated with glyceollins by abiotic and biotic elicitors

**Fig. 5:**
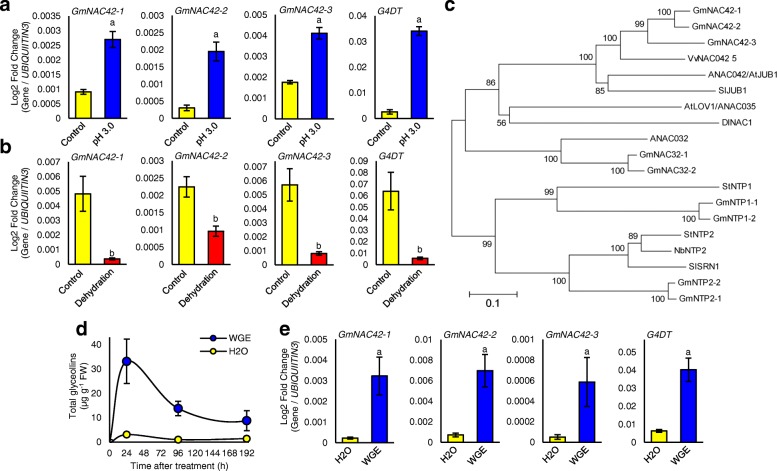
*GmNAC42s* expressions are induced with glyceollins in response to abiotic and biotic elicitors. **a**, **b** Gene expressions following treatment with pH 3.0 medium at 9 dat or dehydration at 6 dat relative to their respective controls measured by qRT-PCR. ^a^Significantly greater and ^b^significantly less than control, paired students t-test (*P* < 0.01). **c** Unrooted phylogenetic tree of GmNAC42 amino acid sequences and characterized NACs. GenBank Accessions and Phytozome landmarks: GmNAC42–1 (KRH73619; Glyma.02G284300), GmNAC42–2 (KRH14512; Glyma.14G030700), GmNAC42–3 (KRG98971; Glyma.18G110700), VvNAC042_5 (XP_002283251; VIT_12s0028g00860), ANAC042 (Q9SK55; AT2G43000), SlJUB1 (XP_019069297; Solyc05g021090); AtLOV1/ANAC035 (Q9ZVP8.2; AT2G02450), DlNAC1 (ABQ96120; N/A), ANAC032 (AAM65083; AT1G77450), GmNAC32–1 (NP_001236871.2; Glyma.06G114000), GmNAC32–2 (NP_001240958.1; Glyma.04G249000), StNTP1 (AGY49284; PGSC0003DMT400001498), StNTP2 (AGY49285; PGSC0003DMT400079997), NbNTP2 (AGY49287; N/A), SlSRN1 (NP_001304297; Solyc12g056790), GmNTP1–1 (NP_001276287; Glyma.02G222300), GmNTP1–2 (XP_006596412; Glyma.14G189300), GmNTP2–1 (XP_003556180; Glyma.20G172100), GmNTP2–2 (NP_001274375; Glyma.10G219600). The number adjacent branches indicate maximum parsimony bootstrap values for the corresponding node. The scale bar indicates the number of differences per 100 residues derived from the Muscle alignment. The phylogenetic tree was generated using MEGA v5.0 software [77]. **d** Amount of total glyceollins extracted from empty-vector transformed soybean hairy roots following treatment with H2O or wall glucan elicitor (WGE) from *P. sojae*. **e** Gene expressions following 24 h of WGE or water treatment of soybean hairy roots measured by qRT-PCR

We conducted qRT-PCR to gain insight into whether the NAC42-type TFs that were identified by our transcriptomics analysis may be involved in regulating glyceollin biosynthesis. qRT-PCR confirmed that the three GmNAC42s were upregulated by pH 3.0 medium and downregulated by dehydration (Fig. 5a-b).

The predicted GmNAC42 proteins were 68.5–85.8% similar to each other and 54.3–56.7% similar to ANAC042/JUB1 with GmNAC42–1 being the most similar (Additional file 6: Table S6). The N-terminal halves of these proteins contained the conserved NAM domain (pfam02365) putatively involved in dimerization and binding DNA (Additional file 7: Fig. S1). The N-terminal halves of the GmNAC42s were highly similar to ANAC042/JUB1 (76.2–83.3%), whereas the C-terminal halves putatively involved in protein-protein interactions were highly divergent (30.5–34.9% similarity) (Additional file 6: Table S6). A phylogenetic analysis of the predicted GmNAC42 proteins with characterized NACs revealed that the GmNAC42s were most closely related to VvNAC42_5 (Fig. 5c). *VvNAC42_5* is an SA-independent powdery mildew responsive gene from grapevine (*Vitis vinifera*) [50]. Also in this cluster were proteins that positively regulate drought stress responses, namely SlJUB1 and DlNAC1 [51, 52].

To probe further whether *GmNAC42*s may be positive regulators of glyceollins, we assessed whether their gene expressions were upregulated by the wall glucan elicitor (WGE) from *P. sojae*.

Treatment of soybean hairy roots with WGE resulted in maximum accumulation of glyceollins at 24 h after treatment (Fig. 5d). qRT-PCR found that all three *GmNAC42*s were upregulated 9.6- to 14.4-fold at this time with the glyceollin biosynthesis gene *G4DT* (Fig. 5e). *GmNAC42–1* was the most highly upregulated.

### *GmNAC42–1* regulates glyceollin biosynthesis in response to *Phytophthora sojae* WGE

We chose to investigate the function of *GmNAC42–1* since it is the soybean homolog of *ANAC042*, an indole alkaloid phytoalexin regulator from Arabidopsis, and since its gene expressions coincided with the elicitation of glyceollin biosynthesis. If *GmNAC42–1* positively regulates glyceollin biosynthesis, silencing its gene expressions in elicited tissues should reduce the accumulation of glyceollin metabolites and biosynthesis gene transcripts. Conversely, overexpressing *GmNAC42–1* should increase the accumulation of glyceollins and their biosynthesis gene transcripts. To test, we produced soybean hairy roots harboring an RNA interference (RNAi) construct that encoded a hairpin dsRNA identical to a 227 bp region of exon 2 of *GmNAC42–1* and roots that overexpressed the *GmNAC42–1* open reading frame (ORF) via the constitutive cauliflower mosaic virus promoter (p35S).

**Fig. 6:**
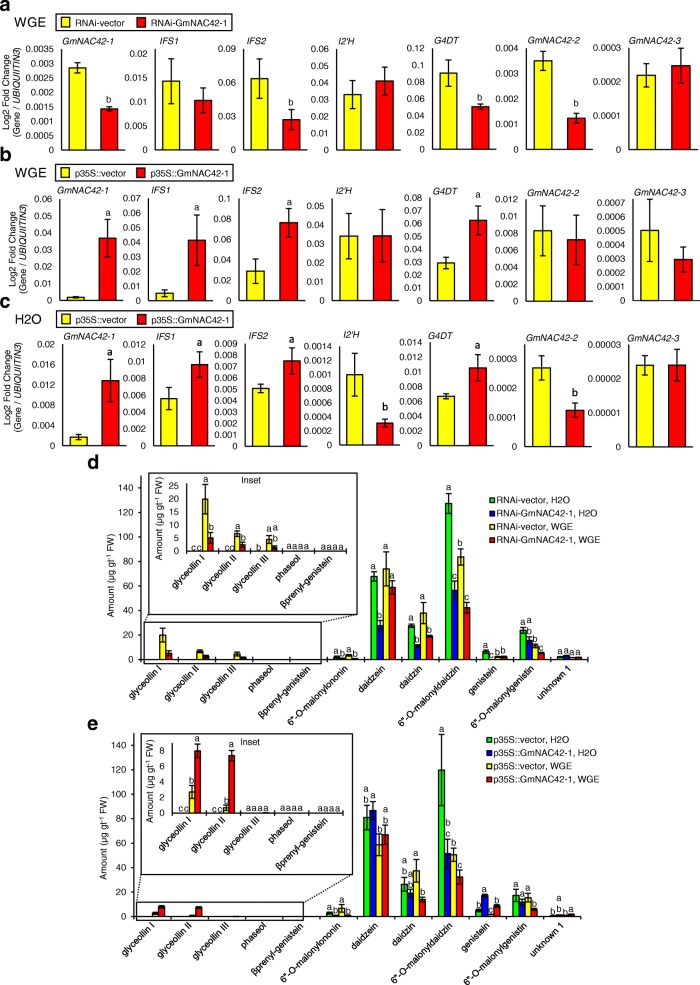
Overexpression and silencing of *GmNAC42–1* in soybean hairy roots. **a** Gene expressions in WGE-treated Williams 82 hairy roots undergoing RNAi silencing of *GmNAC42–1.* **b** Gene expressions in WGE-treated hairy roots overexpressing *GmNAC42–1.* **c** Gene expressions in mock-treated hairy roots overexpressing *GmNAC42–1.* Measurements were 24 h after treatment by qRT-PCR. ^a^Significantly greater and ^b^significantly less than control, paired students *t*-test (*P* < 0.01). **d** Amounts of phytoalexins and constitutive isoflavonoids in soybean hairy roots undergoing RNAi silencing of *GmNAC42–1* 24 h after treatment with WGE or H2O. **e** Metabolite amounts from hairy roots overexpressing *GmNAC42–1.* Different letters show significant differences by single factor ANOVA, Tukey post hoc test, *P* < 0.01

A 2.0-fold silencing of *GmNAC42–1* decreased the accumulations of glyceollin biosynthesis gene transcripts *IFS1*, *IFS2*, and *G4DT* 1.8- to 2.4-fold (Fig. 6a). Off-target silencing of *GmNAC42–2* was observed but not for *GmNAC42–3*. The overexpression of *GmNAC42–1* upregulated *IFS1*, *IFS2*, and *G4DT* from 2.1- to 8.3-fold in roots treated with WGE or mock (H_2_O) (Fig. 6b-c).

RNAi silencing of WGE-elicited roots decreased the amounts of glyceollin I, II and III 4.0-, 2.8- and 3.2-fold, respectively (Fig. 6d). It also caused 2.0-fold decreases in the amounts of 6-*O*-malonyldaidzin and daidzin (Fig. 6d), consistent with decreased expressions of *IFS2* (Fig. 6a). Overexpressing *GmNAC42–1* in WGE-elicited roots resulted in 10.8-, 4.9-, and 3.0-fold increases in the amounts of glyceollin I, genistein, and glyceollin II, respectively (Fig. 6e). It also caused a 1.6-to 2.7-fold reduction in the amounts of daidzin and 6-*O*-malonyldaidzin, consistent with upregulating *G4DT* (Fig. 6b). In the absence of WGE treatment, the overexpression of *GmNAC42–1* alone was not sufficient to stimulate glyceollin accumulation, reflecting its inability to upregulate all glyceollin-specific biosynthesis genes when overexpressed. However, it did result in a 2.4-fold reduction in the amounts of 6-*O*-malonyldaidzin [21].

### GmNAC42–1 localizes to the nucleus and directly binds the promoters of glyceollin biosynthesis genes

**Fig. 7:**
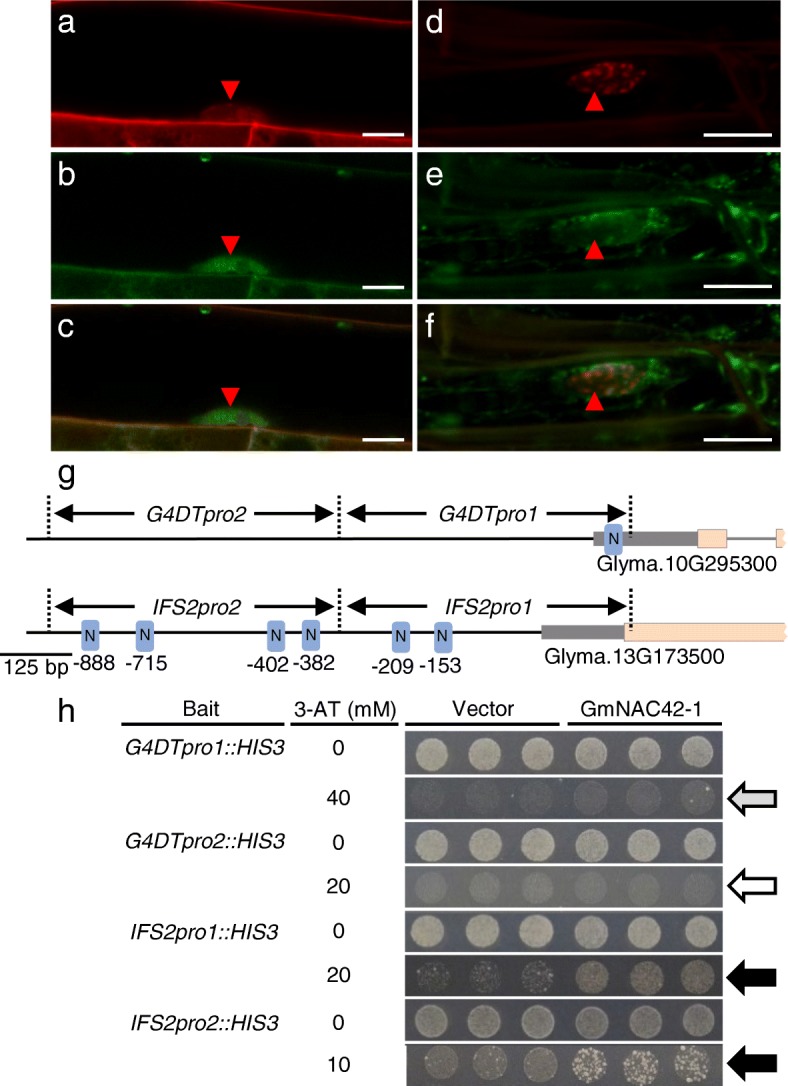
Nuclear localization and DNA binding activities of GmNAC42–1. (**a**-**f**) Confocal fluorescence microscopy images of GFP-GmNAC42–1 fusion protein in transgenic soybean hairy roots. **a**-**c** Root cell expressing GFP-GmNAC42–1 fusion protein. **d**-**f** Root cell expressing GFP. **a** Propidium iodide (10 μg mL^− 1^) staining the plasma membrane and nucleus. **b** GFP-GmNAC42–1 fluorescence at plasma membrane and nucleus. **c** Overlay of GmNAC42–1 and propidium iodide fluorescence from panels **a** and **b**. **d** Propidium iodide staining. **e** GFP signal in cytosol and other extranuclear compartments. **f** Overlay of GFP and propidium iodide fluorescence from D and E. Bars = 10 μm. **g** Schematic diagram demonstrating *G4DT* and *IFS2* promoter fragments used for yeast one-hybrid assays and predicted NAC binding elements (blue boxes). **h** Yeast one-hybrid assays of YM4271 yeast transformed with *GmNAC42–1* on SD/−His/−Leu medium containing various concentrations of 3-AT. Arrows: gray indicates weak binding, white no binding, and black strong binding

To determine whether the subcellular localization of the GmNAC42–1 protein was consistent with its putative role as a TF, we cloned its ORF downstream of an N-terminal GFP tag and expressed the translational fusion in soybean hairy roots using the constitutively active CaMV-35S promoter (p35S) [53]. nGFP-GmNAC42–1 localized to the nucleus as shown by co-localization with propidium iodide fluorescence (red arrowheads, Fig. 7a–c). By contrast, GFP expressed by the empty vector localized to the cytosol and other extra-nuclear compartments (Fig. 7d–f).

To test whether the GmNAC42–1 protein could directly bind the promoters of glyceollin biosynthesis genes, the ORF was also cloned downstream of the GAL4 activation domain and expressed in yeast harboring several 500 bp segments of *IFS2* or *G4DT* promoters (Fig. 7g). GmNAC42–1 weakly activated the *G4DT* promoter segment closest the transcription start site (*G4DTpro1*) that had one predicted NAC binding element (T/ATTGACT/C), failed to activate the segment that lacked the element (*G4DTpro1*), and strongly activated both *IFS2* promoter segments that each had several elements (Fig. 7h).

## Discussion

### *GmNAC42–1* is required for full elicitation of glyceollin biosynthesis

In this study, we found that transcripts of the NAC-family TF gene *GmNAC42–1* were upregulated with glyceollin biosynthesis genes and metabolites when soybean tissues were elicited by acidity stress or the biotic elicitor WGE from *P. sojae*. They were also downregulated with glyceollin biosynthesis genes and metabolites by dehydration stress. The overexpression and silencing of *GmNAC42–1* in WGE-treated hairy roots enhanced and suppressed, respectively, the expressions of the isoflavone biosynthetic genes *IFS1* and *IFS2*, the glyceollin-specific gene *G4DT*, and the accumulation of glyceollin metabolites. Since *G4DT* is specifically involved in glyceollin biosynthesis, the results suggest that *GmNAC42–1* is a regulator of glyceollin elicitation and not the biosynthesis of constitutively accumulating isoflavone conjugates. However, overexpressing or silencing *GmNAC42–1* did not affect the expression levels of *I2’H*, one of the key genes required for glyceollin biosynthesis [54]. Further, overexpression of *GmNAC42–1* in the absence of WGE did not result in the accumulation of glyceollins. Thus, our results showed that *GmNAC42–1* is required for the full elicitation of glyceollin biosynthesis in response to *P. sojae* WGE, but is not sufficient to upregulate all glyceollin biosynthesis genes.

The nGFP-GmNAC42–1 fusion protein localized to the nucleus in the absence of an elicitor treatment and thus did not rely on elicitor treatment for nuclear localization as observed for the phytoalexin TF AtWRKY33 or the NAC-family TFs StNTP1 and StNTP2 [55, 56]. Since *GmNAC42–1* is essential for full elicitation of glyceollins, we suggest that *GmNAC42–1* acts in concert with at least one other TF to coordinately activate all glyceollin biosynthetic genes. Further, by upregulating some but not all glyceollin genes, *GmNAC42–1* could also function in SAR to prime soybean tissues distal to an inoculation site for subsequent rapid/high-level elicitation [23, 57, 58]. A subsequent direct inoculation of the primed tissues would activate the expressions or activity of one or more additional TFs that upregulates *I2’H* and other glyceollin biosynthesis genes that are not regulated by *GmNAC42–1* alone. In that case, overexpressing *GmNAC42–1* could serve as an alternative to spraying the lactofen-containing herbicide Cobra that primes glyceollin biosynthesis to increase resistance against pathogens such as white mold, the causal agent of sclerotinia stem rot, without adversely effecting yield [59, 60]. Future experiments should test whether overexpressing *GmNAC42–1* in soybean plants primes glyceollin biosynthesis without adverse effects on yield as well. Since the rapidity of glyceollin elicitation is a major factor that distinguishes *resistant to* *P*. *sojae* (Rps) soybean genotypes from nearly-isogenic susceptible genotypes [61–64], experiments should also test whether overexpressing *GmNAC42–1* enhances the rapidity of glyceollin elicitation in response to compatible *P.* *sojae* (Rps) genotypes.

### *GmNAC42–1* and a conserved phytoalexin elicitation pathway

The regulation of phytoalexins by pathogens and specific abiotic stresses suggests that elicitation is highly complex and may require multiple signaling pathways. This study in soybean identified acidity stress (pH 3.0 medium) and dehydration as novel regulators of phytoalexin biosynthesis. Transcriptome analysis found that the genes upregulated by acidity stress and downregulated by dehydration were reminiscent of pathogen responses, with SAR genes being highly overrepresented. The SAR genes included homologs of Arabidopsis *ALD1* and *FMO1* that synthesize the systemic signaling molecules Pip and its derivative N-hydroxypipecolic acid (NHP) to orchestrate priming of pathogen responses [46, 47], and the lipase-like and TF Arabidopsis genes *PAD4* and *ANAC042* that regulate the biosynthesis of camalexin in Arabidopsis [7, 48]. Here, we found that *GmNAC42–1* is the soybean homolog of *ANAC042* and is required for full elicitation of glyceollins. The results suggest a conserved phytoalexin elicitation pathway for phenylpropanoid-derived glyceollins in soybean and indole alkaloid-derived camalexin in Arabidopsis that requires NAC42 TFs. Further, our investigation of Lager’s transcriptome dataset [65] demonstrated that *ANAC042* and its target camalexin biosynthesis genes (namely *CYP71A12, CYP71A13* and *CYP71B15/PAD3*) [7] were upregulated by long-term acidity stress, suggesting that NAC42-dependent induction of phytoalexins may be a conserved response to acidity stress.

More insight into the NAC42 pathway could be drawn from the fact that glyceollin biosynthesis was elicited by the treatment of soybean cotyledons with hydroxyl radical (a ROS) [24] and camalexin elicitation by the ROS-inducing herbicide acifluorofen required *ANAC042* [7]. The ROS-inducing herbicide lactofen systemically primes glyceollin biosynthesis [59]. ROS accumulation is stimulated by various phytoalexin elicitors such as pathogens, heavy metals, and UV irradiation [66–68]. Further, the acidification of growth media from pH 5.0 to 4.5 stimulated ROS production in seedlings of barley and Scots pine [69, 70] and in MS medium containing Plantago shoots [71]. Also, genes that positively regulate ROS (GO:2000377 and GO:2000379) were overrepresented in the soybean and Arabidopsis transcriptome responses to long-term acidity stress. Thus, the NAC42 pathway may be a conserved ROS signaling pathway responsible for phytoalexin elicitation in response to various abiotic and biotic elicitors. It is tempting to speculate that major TFs that regulate acidity and dehydration responses may regulate *GmNAC42–1* since the stresses oppositely regulate *GmNAC42–1* transcripts. *STOP1* is a zinc finger TF that is a major regulator of protective responses to acidity stress [72, 73]. STOP1 also stimulates ROS production [74]. Yet, *STOP1* homologs were not found in the soybean transcriptome response to long-term acidity stress (9 dat), and *ANAC042* was not downregulated in an Arabidopsis *stop1* mutant at 1 dat [72]. This could infer that *NAC42* induction of phytoalexins is downstream of ROS signaling and not directly regulated by *STOP1*. ABA is a major regulator of dehydration responses in part through the activity of ABA-responsive element (ABRE)-binding TFs [75]. Our transcriptome dataset shows that dehydration is a powerful negative regulator of glyceollin biosynthesis and *GmNAC42–1*, raising the possibility that both are negatively regulated by ABA. We found that ABREs were present in the promoter regions (~ 1000 bp upstream of the transcription start site) of several glyceollin biosynthesis genes, but no ABREs were observed in the *GmNAC42–1* promoter (data not shown). Thus, dehydration may regulate glyceollin biosynthesis at multiple levels.

### Co-option of phytoalexin biosynthesis by NAC42

Phytoalexin TF genes of the NAC, MYB, bHLH, and WRKY families have been identified from Arabidopsis, rice, cotton, maize and grapevine [5–10]. Yet none of these TF genes were homologous among plant species. The phytoalexins elicited in these species were biosynthetically diverse and included indole alkaloids, momilactones and phytocassanes, terpenoid aldehydes, deoxyanthocyanidins, and stilbenoids, respectively. Thus, it has remained a question whether any phytoalexin TFs are conserved in plants or whether they are as diverse as the biosynthetic pathways that they regulate. Here, we found that *GmNAC42–1* is required for the full activation of glyceollin biosynthesis in soybean. Its homolog *ANAC042* is needed for the full elicitation of camalexin biosynthesis in Arabidopsis [7]. Glyceollins are isoflavonoid derivatives derived from phenylalanine, whereas camalexin is an indole alkaloid biosynthesized from tryptophan. It is possible that NAC42 TFs regulate genes in the shikimate pathway that produces phenylalanine and tryptophan. Yet, our overexpression and silencing experiments demonstrated that *GmNAC42–1* regulated isoflavonoid- and glyceollin-specific biosynthetic genes through the direct binding of their promoters. While our promoter sequence analyses identified the putative NAC-binding element T/ATTGACT/C within 1 kb of the translation start sites of the camalexin-specific biosynthetic genes *CYP71A12* and *CYP71A13* that were regulated at the mRNA level by *ANAC042* [7], the DREB2A element that was suggested to be the target of ANAC042/JUB1 [76] was not found in those regions nor within glyceollin biosynthetic gene promoters. If NAC42 TFs indeed bind the element T/ATTGACT/C element in glyceollin- and camalexin-specific biosynthetic genes, this would suggest that phytoalexin biosynthesis pathways were co-opted into stress-inducible regulation by NAC42 TFs. Our future work will focus on characterizing the recognition elements and DNA binding domains of GmNAC42–1 and ANAC042 that are required to activate phytoalexin biosynthesis.

## Conclusions

GmNAC42–1 is essential for the full elicitation of glyceollins in soybean. It’s overexpression in elicited soybean hairy roots enhanced the biosynthesis of glyceollins more than 10-fold. Thus, bioengineering the expressions of GmNAC42–1 may be a promising approach for bioproducing glyceollins for medicinal use or for enhancing soybean resistance to the economically destructive pathogen *P. sojae*. GmNAC42–1 is the first identified conserved regulator of phytoalexin biosynthesis and is a homolog of the indole alkaloid phytoalexin regulator *ANAC042* from Arabidopsis. Possible implications are that NAC42-type TF genes could be used in a wide variety of crop plants to enhance the bioproduction of medicinal metabolites or for improving crop resistance to pathogens.

## Additional files

Additional file 1: **Table S1.** Sequences of primers used in the experiments. (XLSX 11 kb)

Additional file 2: **Table S2.** Sequences of promoters used in yeast one-hybrid experiments. (XLSX 12 kb)

Additional file 3: **Table S3.** Genes upregulated by pH 3.0 medium compared to control at 9 dat in Harosoy 63 soybean seedlings. (XLSX 193 kb)

Additional file 4: **Table S4.** Genes downregulated by dehydration in Harosoy 63 soybean seedlings compared to the control at 6 days after treatment. (XLSX 503 kb)

Additional file 5: **Table S5.** Genes upregulated by pH 3.0 medium compared to control at 9 dat and downregulated by dehydration compared to control at 6 dat in Harosoy 63 soybean seedlings. (XLSX 105 kb)

Additional file 6: **Table S6.** Amino acid similarities of NAC42 proteins from soybean, Arabidopsis and grapevine. Full-length proteins (N-terminal and C-terminal halves). (XLSX 11 kb)

Additional file 7: **Figure S1.** Amino acid alignment of NAC42 proteins from soybean, Arabidopsis and grapevine. (DOCX 14 kb)

## Abbreviations

ABRC: Arabidopsis Biological Resource Center
ABRE: ABA-responsive element
AgNO3: silver nitrate
ALD1: AGD2-LIKE DEFENSE RESPONSE PROTEIN 1
aos: allene oxide cyclase
CAD1: (+)-δ-cadinene synthase
CUC2: Cup-shaped cotyledon
CuCl2: Copper chloride
DMAPP: Dimethylallyl diphosphate
DW: Dry weight
FDR: False discovery rate
FMO1: FLAVIN-DEPENDENT-MONOOXYGENASE1
FW: Fresh weight
G4DT: Glycinol:4-dimethylallyl diphosphate transferase
G4DTpro1: G4DT gene promoter segment1
GC: Germination and co-cultivation
GRN: Gene regulatory network
HRG: Hairy root growth
MeJA: Methyl jasmonate
MEP: Methylerythritol phosphate
MS: Murashige and Skoog
NAM: No apical meristem
nGFP: N-terminal GFP tag
NHP: N-hydroxypipecolic acid
PAD4: PHYTOALEXIN DEFICIENT4
PAL: Phenylalanine ammonia lyase
PaNie: *Pythium aphanidermatum*
Pip: Pipecolic acid
qRT-PCR: quantitative reverse transcriptase-polymerase chain reaction
RIN: RNA Integrity Number
RNAi: RNA interference
RNA-seq: RNA sequencing
ROS: Reactive oxygen species
Rps: Resistant to P. sojae
SAR: Systemic acquired resistance
SD-His: Synthetic dropout medium lacking histidine
SIB1: SIGMA FACTOR BINDING PROTEIN 1
STS: STILBENE SYNTHASE
TF: Transcription factor
WGE: Wall glucan elicitor
Y1H: Yeast one-hybrid
3-AT: 3-amino-1,2,4-triazole
ATAF1/2: Arabidopsis transcription activation factor
NAC: NAM/ATAF1/2/CUC2
osjar1–2: *Oryza sativa* jasmonic acid-amido synthetase
