# Supplementary material for: The NAC family transcription factor GmNAC42–1 regulates biosynthesis of the anticancer and neuroprotective glyceollins in soybean
Source: BMC Genomics. 2019 Feb 20;20:149. doi: 10.1186/s12864-019-5524-5 (PMC6381636; doi:10.1186/s12864-019-5524-5)
Supplement: Supplementary file 7 — Figure S1. Amino acid alignment of NAC42 proteins from soybean, Arabidopsis and grapevine. (DOCX 14 kb) [file 12864_2019_5524_MOESM7_ESM.docx]

GmNAC42-1 --------------MDVAKLHNSDDDDDKKEDEDVLPGFRFHPTDEELVGFYLRRKVENK 46

GmNAC42-2 MKVPKFSSVAHRLIMDVAKLHNSDDDDDKKEEEVVLPGFRFHPTDEELVGFYLRRKVEKK 60

GmNAC42-3 -----------------MNVKVHSSDDTKKDEEVVLPGFRFHPTDEELVGFYLQRKVDKK 43

VvNAC042_5 --------------MEVEKTS-TLDNKKDDDEEVVLPGFRFHPTDEELVGFYLRRKVEKK 45

ANAC042 -----------------MSGEGNLGKDHEEENEAPLPGFRFHPTDEELLGYYLRRKVENK 43

. ... ..::* *************:*:**:***::*

GmNAC42-1 PLRIELIKQIDIYKYDPWDLPKVS-SVGEKEWYFFCIRGRKYRNSIRPNRVTGSGFWKAT 105

GmNAC42-2 PLRIELIKQIDIYKYDPWDLPKVS-SVGEKEWYFFCIRGRKYRNSIRPNRVTGSGFWKAT 119

GmNAC42-3 PLKIELIKQVDIYKYDPWDLPKVN-SFGDKEWYFFCIRGRKYRNSVRPNRVTRSGFWKAT 102

VvNAC042_5 PISLELIKQVDIYKYDPWDLPRVSTNMGDREWYFFCIRGRKYRNSIRPNRVTGSGFWKAT 105

ANAC042 TIKLELIKQIDIYKYDPWDLPRVS-SVGEKEWYFFCMRGRKYRNSVRPNRVTGSGFWKAT 102

: :*****:***********:*. ..*::******:********:****** *******

GmNAC42-1 GIDKPIYCVKEPHECIGLKKSLVYYRGSAGKGTKTDWMMHEFRLPPNGKTSNNPQAND-- 163

GmNAC42-2 GIDKPIYCVREPQECIGLKKSLVYYRGSAGKGTKTDWMMHEFRLPPNGKTSNNPQANDAN 179

GmNAC42-3 GIDKSIYCVKEPHECIGLKKSLVYYRGSAGKGTKTDWMMHEFRLPPNAKSS-------PN 155

VvNAC042_5 GIDKPIYSVEATNECIGLKKSLVYYRGSAGKGTKTDWMMHEFRLPPGGKGTYFPNA--KN 163

ANAC042 GIDKPVYSN---LDCVGLKKSLVYYLGSAGKGTKTDWMMHEFRLPSTTKTD--------S 151

**** :*. :*:********* ******************* *

GmNAC42-1 -AQEAEVWTLCRILKRFPSYKKYTPNLKDSAAAPIMTKPNYPTNSSSTSKTCSLESD-NG 221

GmNAC42-2 DVQEAEVWTLCRIFKRVPTYKKYTPNLKDSAAP--ITEPNHPTNS-SSSKTCSLESD-NC 235

GmNAC42-3 DVQEAEVWTLCRIFKRIPSFKKYAPNLKDSTPATK---PN-PVN----SNASTLDSDNCC 207

VvNAC042_5 VTQEAEVWTLCRIFKRSPTYRKYTPEGKGSISN-------KQVATDSSSKTCSLESDNND 216

ANAC042 PAQQAEVWTLCRIFKRVTSQRNPTILPPNRKPV--------ITLTDTCSKTSSLDSDHTS 203

.*:*********:** : :: : . . *::.:*:**

GmNAC42-1 KPYLTFTNSLSPLLIQQNERKPAINGHVDVERNHLFLGQLGINV-AQAPTSLSYSSFWNR 280

GmNAC42-2 KPYLTFTNSSSPQLILQNERKPVINGHVDVERNHLFLGQLGNVA--------QAPSFWNH 287

GmNAC42-3 KPYLIC---RDPMMMQQIERKPVIGQV--DEKKHLFLGQFGPLSHLQAPSTTPYPNFWNQ 262

VvNAC042_5 -KYLSFENSTTRTK-----NSTVVDS-SIDERSQLVVGQM---SSFVSPYSGPYSSFWNP 266

ANAC042 HRTVDSMSHEPPL---PQPQNPYWNQHIVGFNQP----------TYTGNDNNLLMSFWNG 250

: .. . .. .***

GmNAC42-1 HHQNNVEFANENWDDLTSVVQFANIDPSRVSDHCKEFNRF--- 320

GmNAC42-2 HQNNNVEFANENWDDLTSVVQFA-IDPSRVSDHSKEFNRF--- 326

GmNAC42-3 NNVEDHAFANENWDDLRSVVQFA-IDPSTVFDCTETYIPPYFS 304

VvNAC042_5 NGN-DQFFTSGNWDELRSIVESA-IDPPHVYDCR--------- 298

ANAC042 NGG-DFIGDSASWDELRSVIDGNT-KP---------------- 275

: : . .**:* *::: .*

**Additional file 7: Figure S1.** Amino acid alignment of NAC proteins. Residues that are fully conserved (*), strongly similar (:) (> 0.5 in the Gonnet PAM 250 matrix), and weakly similar (.) (Gonnet PAM <0.5 and > 0). NAM domain is underlined. N-terminal half is shaded.
